# Identification of New Delhi Metallo-β-lactamase 1 in *Acinetobacter lwoffii* of Food Animal Origin

**DOI:** 10.1371/journal.pone.0037152

**Published:** 2012-05-18

**Authors:** Yang Wang, Congming Wu, Qijing Zhang, Jing Qi, Hebing Liu, Yu Wang, Tao He, Licai Ma, Jing Lai, Zhangqi Shen, Yuqing Liu, Jianzhong Shen

**Affiliations:** 1 Key Laboratory of Development and Evaluation of Chemical and Herbal Drugs for Animal Use, College of Veterinary Medicine, China Agricultural University, Beijing, People's Republic of China; 2 Department of Veterinary Microbiology and Preventive Medicine, College of Veterinary Medicine, Iowa State University, Ames, Iowa, United States of America; 3 Institute of Animal Science and Veterinary Medicine, Shandong Academy of Agricultural Sciences, Jinan, People's Republic of China; Institut National de la Recherche Agronomique, France

## Abstract

**Background:**

To investigate the presence of metallo-β-lactamase (MBL) genes and the genetic environment of the New Delhi metallo-β-lactamase gene *bla*_NDM-1_ in bacteria of food animal origin.

**Methodology/Principal Findings:**

Gram-negative bacteria with low susceptibility to imipenem (MIC>8 µg/mL) were isolated from swab samples collected from 15 animal farms and one slaughterhouse in eastern China. These bacteria were selected for phenotypic and molecular detection of known MBL genes and antimicrobial susceptibility testing. For the *bla*_NDM-1_ positive isolate, conjugation and transformation experiments were carried out to assess plasmid transfer. Southern blotting was conducted to localize the *bla*_NDM-1_ genes, and DNA sequencing was performed to determine the sequences of *bla*_NDM-1_ and the flanking genes. In total, nine Gram-negative bacteria of four different species presented a MBL phenotype. *bla*_NDM-1_ was identified on a mobile plasmid named pAL-01 in an *Acinetobacter lwoffii* isolate of chicken origin. Transfer of pAL-01 from this isolate to *E. coli* J53 and JM109 resulted in resistance to multiple β-lactams. Sequence analysis revealed that the *bla*_NDM-1_ gene is attached to an intact insertion element IS*Aba125*, whose right inverted repeat (IR-R) overlaps with the promoter sequence of *bla*_NDM-1_. Thus, insertion of IS*Aba125* likely enhances the expression of *bla*_NDM-1_.

**Conclusion:**

The identification of a *bla*_NDM-1_- carrying strain of *A. lwoffii* in chickens suggests the potential for zoonotic transmission of *bla*_NDM-1_ and has important implications for food safety.

## Introduction

Metallo-β-lactamases (MBLs) in clinical Gram-negative organisms are an important threat to public health. MBLs, which require divalent cations (usually zinc ions) as metal cofactors for enzymatic activity, can hydrolyze all β-lactams including carbapenems (except aztreonam) [1]. In 2009, a novel MBL enzyme, named New Delhi metallo-β-lactamase (NDM-1, encoded by *bla*_NDM-1_), was identified in plasmids from *Klebsiella pneumoniae* and *Escherichia coli* isolates recovered from a Swedish patient previously hospitalized in India [2]. Recently, *bla*_NDM-1_-harboring strains of *Enterobacteriaceae* have been identified worldwide, with most reports indicating that the isolates originated from the Indian subcontinent with hospital or community acquisition [3] or from the Balkan countries [4]. However, NDM-1 positive strains have also been reported in patients with no known contact with these areas [5]. Coexistence of *bla*_NDM-1_ with the class D carbapenemase gene *bla*_OXA-23_ in clinical isolates of *Acinetobacter baumannii* was first detected in India [6]. Subsequently, five *bla*_NDM-1_-harboring *A. baumannii* clinical strains were reported in five different provinces in China [7], [8]. A multidrug resistant *Acinetobacter lwoffii* strain carrying a *bla*_NDM-1_ plasmid was also isolated from the urine of a female patient in China [9]. Although China borders India and Pakistan, there was no evidence that the infected patients had any contact with the Indian subcontinent.

Recently, *bla*_NDM-1_ was found in a number of bacterial species isolated from water and seepage samples in central New Delhi, suggesting it is widely disseminated in the local environment [10]. In addition, *bla*_NDM-1_-carrying bacteria have also been reported as gut colonizers in patients with no clinical symptoms, suggesting the possible transmission of *bla*_NDM-1_ via the fecal-oral route, and that fecal flora may serve as a reservoir for *bla*_NDM-1_ dissemination [3], [10], [11]. These findings suggest that the spread of the NDM-1 resistance determinant is much wider than previously realized. However, no information is available on the distribution of NDM-1 in food producing animals. Here we investigated the prevalence of Gram-negative bacteria with low susceptibility to imipenem in food producing animals, and determined whether MBL genes, including *bla*_NDM-1_, were present in these animal isolates.

## Results

### Prevalence of Gram-negative bacteria with low susceptibility to imipenem in food producing animals

In total, nine isolates with very low susceptibility to imipenem (MIC>8 µg/mL for imipenem), obtained from eight of the 396 samples, were confirmed as Gram-negative bacteria. The isolates were identified by ATB 32 GN, ATB 32E and 16 S rDNA sequencing analysis as *Stenotrophomonas maltophilia* (n = 3), *Chryseobacterium indologenes* (n = 3), *Myroides odoratimimus* (n = 1), *Myroides odoratus* (n = 1), and *A. lwoffii* (n = 1). Eight of these isolates came from pig samples, while the *A. lwoffii* isolate was derived from a chicken sample. No imipenem-resistant *Enterobacteriaceae* strains were identified in this study. Two isolates, *M. odoratimimus* LYP-BCYg6a and *M. odoratus* LYP-BCYg6b, were collected from one rectal swab sample from a pig farm.

### Characterization of the nine Gram-negative bacteria with low susceptibility to imipenem

All nine Gram-negative isolates presented high MIC values for three tested carbapenems, including imipenem (ranging from 32–128 µg/mL), meropenem (16–64 µg/mL), and ertapenem (16–512 µg/mL). Imipenem-EDTA double-disc synergy tests and E-test MBL strip tests confirmed that all nine isolates were MBL-positive. However, PCR analysis using primers specific for known mobile and chromosomal MBL genes revealed that only the *A. lwoffii* strain SGC-HZ9, of chicken origin, was *bla*_NDM-1_ positive. This *bla*_NDM-1_-harboring strain was resistant not only to all tested β-lactams (except aztreonam, 8 µg/mL) but also to ciprofloxacin (8 µg/mL), tetracycline (32 µg/mL), kanamycin (64 µg/mL), and chloramphenicol (32 µg/mL), while remaining susceptible to gentamicin (0.5 µg/mL) and polymyxin B (0.016 µg/mL) (Table 1). In addition, two ubiquitous free-living bacteria (one *Myroides odoratimimus* LYP-BCYg6a and one *Myroides odoratus* isolate LYP-BCYg6b) harbored a chromosomal MBL gene, *bla*_MUS-1_. Six isolates with a MBL phenotype did not contain any of the tested MBL genes.

**Table 1:** Antimicrobial susceptibility patterns of *A. lwoffii* SGC-HZ9, transformants and transconjugants.

Antimicrobial agents^a^	MIC (µg/mL)				
	*A. lwoffii* SGC-HZ9	*E. coli* J53	*E. coli* J53 (pAL-01)	*E. coli* JM109	*E. coli* JM109 (pAL-01)
IMP	32	1	4	0.25	1
MER	64	0.03	1	0.016	1
ERT	512	0.03	2	0.016	2
AMP	32	0·5	32	1	128
CRA	>512	16	256	16	256
COT	>512	0.06	16	0.125	32
CAZ	>512	0.125	256	0.25	>256
CEP	128	0.06	1	0.016	2
AZT	8	0.06	0.125	0.125	0.125
POL	0.016	0.25	0.5	0.125	0.25
CIP	8	0.008	0.004	0.06	0.03
TET	32	4	4	4	4
KAN	64	1	1	0.5	0.5
GEN	0.5	0.5	0.5	0.5	0.25
CHL	32	2	2	2	2

a IMP, Imipenem; MER, Meropenem; ERT, Ertapenem; AMP, Ampicillin; CRA, Cefradine; COT, Cefotaxime; CAZ, Ceftazidime; CEP, Cefepime; AZT, Aztreonam; POL, Polymyxin B; CIP, Ciprofloxacin; TET, Tetracycline; KAN, Kanamycin; GEN, Gentamicin; CHL, Chloramphenicol.

### Phenotypic and plasmid characterization of *A. lwoffii* SGC-HZ9

**Figure 1:**
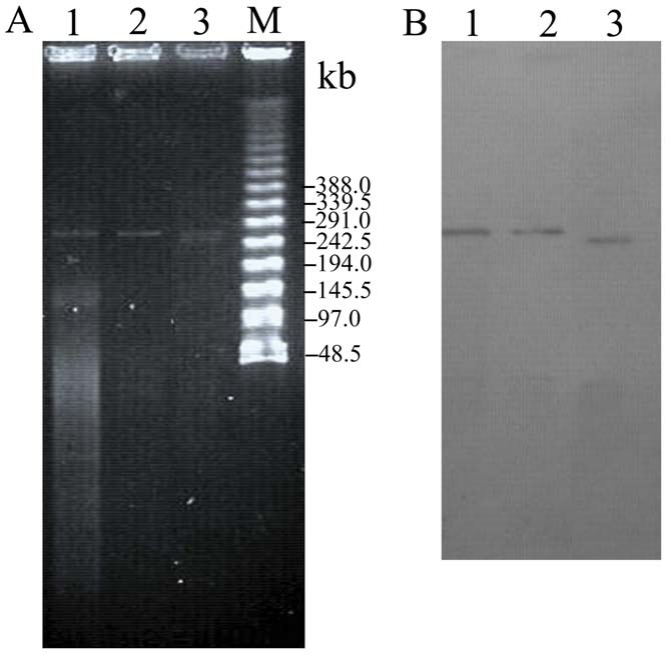
Identification of a mobile *bla*_NDM-1_ gene. (A) PFGE analysis of the plasmid preparations. (B) Southern hybridization of the PFGE gel and S1 nuclease PFGE gel with a *bla*_NDM-1_ probe. For both panels, lanes 1–3 contain plasmids prepared from *A. lwoffii* SGC-HZ9, transformant JM109-SGC-HZ9, and transconjugant J53-SGC-HZ9, respectively. M: Lambda Ladder PFG Marker (NEB, UK).

Plasmids of *A. lwoffii* SGC-HZ9 were extracted and separated by pulsed field gel electrophoresis (PFGE). Subsequent Southern blotting analysis of the PFGE gel revealed hybridization of a *bla*_NDM-1_-specific probe to a ∼270 kb plasmid, which was designated pAL-01 (Figure 1). Plasmid pAL-01 was successfully transferred from SGC-HZ9 to *E. coli* J53 using filter mating and to *E. coli* JM109 using electroporation. The size of the plasmid recovered from transformant JM109-SGC-HZ9 was consistent with that observed using PFGE, while two plasmid bands were identified in transconjugant J53-SGC-HZ9 (approximately 260 kb and 270 kb) (Figure 1A). Southern blotting using the *bla*_NDM-1_ probe further confirmed the presence of pAL-01 in transformant JM109-SGC-HZ9, while in transconjugant J53-SGC-HZ9, the probe hybridized to a 260 kb plasmid, suggesting unknown rearrangements had been occurred in the transconjugants with respect to pAL-01 (Figure 1B). Susceptibility tests revealed that both the transconjugant and transformant presented resistance or decreased susceptibility to all tested β-lactams (except aztreonam) as compared with the recipients *E. coli* J53 and JM109, but remained sensitive to other classes of antibiotics (kanamycin, ciprofloxacin, tetracycline, and chloramphenicol) (Table 1). After 14 passages in media with or without 16 µg/mL of ceftazidime, *A. lwoffii* SGC-HZ9 and the transformant JM109-SGC-HZ9 stably maintained the *bla*_NDM-1_-containing plasmid. However, transconjugant J53-SGC-HZ9 lost *bla*_NDM-1_ within three passages when grown in antibiotic-free media, but maintained *bla*_NDM-1_ in the presence of ceftazidime.

### Genetic sequence flanking *bla*_NDM-1_ in pAL-01

**Figure 2:**
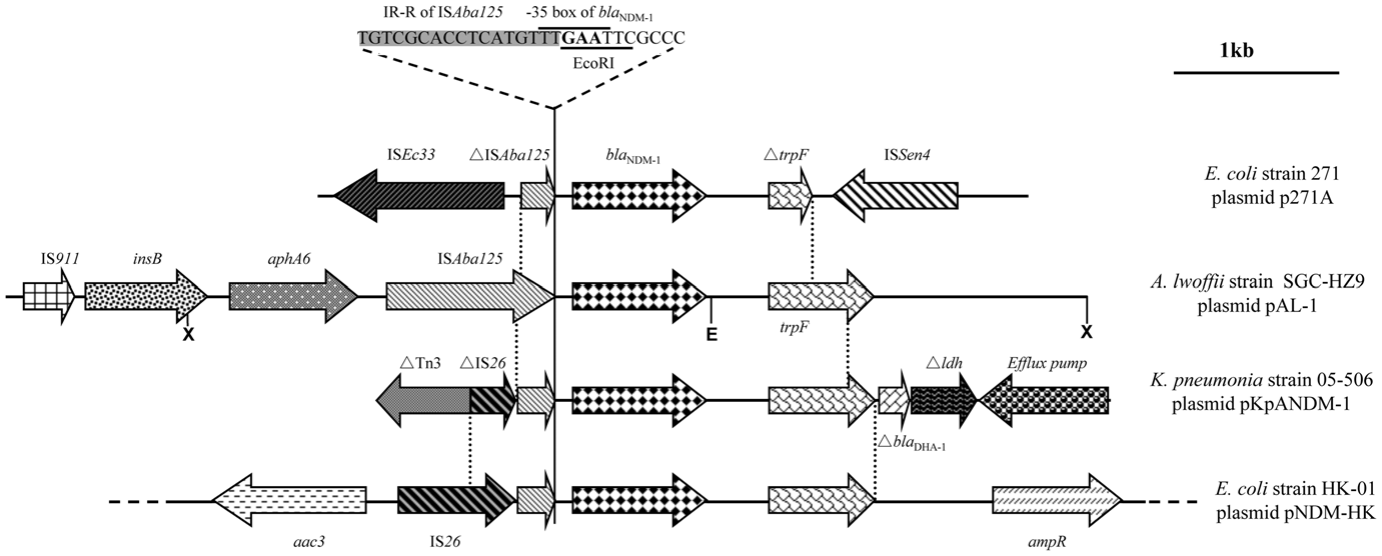
Schematic map representing *bla*_NDM-1_ and its flanking genetic sequences. Comparison of plasmid regions around the *bla*_NDM-1_ gene in *A. lwoffii* SGC-HZ9 (JN616388), *E. coli* 271(HQ162469) [12], *K. pneumoniae* 05-506 (FN396876) [2], and *E. coli* HK-01 (HQ451074) [13]. The vertical dotted lines indicate the locations at which the sequences diverge. X and E depict *Xba*I and *Eco*RI sites, respectively. The −35 promoter region of *bla*_NDM-1_ and the overlapping *Eco*RI site are indicated on top of the panel. The right inverted repeat of IS*Aba125* is shaded and partly overlaps with the −35 box of *bla*_NDM-1_.

Following DNA cloning and modified random primer sequence walking analysis, a 6.5 kb region surrounding the *bla*_NDM-1_ gene (GenBank Accession No. JN616388) was obtained. The gene region included two antimicrobial resistance genes (*bla*_NDM-1_ and *aphA6*), three mobile elements (IS*911*, *insB*, and IS*Aba125*), and phosphoribosylanthranilate isomerase gene *trpF*. A comparison of the regions surrounding *bla*_NDM-1_ in the plasmids of *E. coli* p271A, *A. lwoffii* pAL-01, *K. pneumoniae* pKpANDM-1, and *E. coli* pNDM-HK is presented in Figure 2. The 2022 bp *bla*_NDM-1_-containing region in pAL-01 shared 99% sequence identity with the corresponding region in pKpANDM-1 from *K. pneumoniae* 05-506 (GenBank no. FN396876), with an additional 24 bp inserted into the region between *bla*_NDM-1_ and *traF* in pAL-01. This additional 24 bp was also found in pA271A and pNDM-HK [12], [13].

An intact insertion sequence (IS) element, IS*Aba125*, flanked by imperfect terminal repeats (IR-L AAACTTGAAGTGCGACA, IR-R TGTCGCACCTCATGTTT) was identified immediately upstream of the *bla*_NDM-1_ gene. This IS element belongs to the widely distributed IS*30* family and was previously identified in *A. baumannii* (GenBank no. AY751533), where it is associated with insertional inactivation of an outer-membrane porin [14]. Notably, the *bla*_NDM-1_ upstream regions in pKpANDM-1, pA271A, and pNDM-HK only contained partial IS*Aba125* sequences, along with the terminal repeat IR-R. In these examples, the IS element was truncated by other insertion sequences, including IS*Ec33* in pA271A and IS*26* in pKpANDM-1 and pNDM-HK (Figure 2). Interestingly, the previously confirmed −35 (TTGAAT) *bla*_NDM-1_ promoter sequence [12] overlaps with the inverted repeat IR-R of IS*Aba125*, and the nucleotides TGA in the −35 box form the terminal codon of IS*Aba125* (Figure 2).

Upstream of IS*Aba125* is an open reading frame encoding an aminoglycoside phosphotransferase, which is 98.5% similar to the kanamycin resistance gene *aphA6* in *A. baumannii* (GenBank no. P09885). Further upstream are two adjacent mobile genes that are identical to putative transposase OrfB (namely *insB*) in *A. baumannii* 1656-2 (GenBank no. CP001921), and the IS*3*/IS*911* family transposase previously identified in *A. baumannii* (GenBank no. CP001921). Downstream of *bla*_NDM-1_, the phosphoribosylanthranilate isomerase gene *trpF* (639 bp) shows 100% similarity to the first 581 nucleotides of *trpF* in pNDM-HK and pKpANDM-1 (Figure 2). The TrpF protein shares 65% amino acid similarity with the phosphoribosylanthranilate isomerase of *Erythrobacter* sp. SD-21(ZP_01863115).

## Discussion

Nine Gram-negative non-fermentative bacteria (three *C. indologenes*, three *S. maltophilia*, two *Myroides* spp, and one *A. lwoffii*) with low susceptibility to imipenem were identified in this study. *Acinetobacter* are common, free-living saprophytes found in soil, water, sewage, and animal source foods. It has been reported that *Acinetobacter* species constitute up to 22·7% of the total microflora of chicken carcasses [15]. *A. lwoffii* is one of the most predominant species of *Acinetobacter* involved in economically-important spoilage of foods such as bacon, chicken, eggs, and fish, even when stored under refrigerated conditions or following irradiation treatment [15]. In the past, *Acinetobacter* spp. were regarded as saprophytes of little clinical significance. However, with the introduction of powerful new antibiotics in clinical practice and agriculture, and the use of invasive procedures in hospital intensive care units, antibiotic resistance-related community- and hospital-acquired *Acinetobacter* infections have emerged with increasing frequency [16], [17].

All nine Gram-negative species isolated in this study were positive for a MBL phenotype. However, only four were found to contain known MBL-conferring genes. Importantly, the newly discovered MBL gene *bla*_NDM-1_ was identified in *A. lwoffii* SGC-HZ9, which not only presented high MIC values to nearly all β-lactams, but was also resistant to four other classes of antibiotics (kanamycin, ciprofloxacin, tetracycline, and chloramphenicol). This isolate was derived from the anal swab of a chicken. Although information on antimicrobial therapy for this particular chicken was not available, the antibiotic usage records for the chicken farm indicated that a number of antimicrobial agents, including penicillin, cefotaxime, cefradine, tilmicosin, doxycycline, and neomycin had been used for curing or preventing bacterial infections. To the best of our knowledge, this is the first report of the *bla*_NDM-1_ gene in *A. lwoffii* of food animal origin, which could have a serious public health implication because the species of *A. lwoffii* is a ubiquitous bacterium in foodstuffs, and can be transferred to human by the food chain. In addition, two *Myroides* spp. isolates derived from the same anal swab in a pig farm harbored *bla*_MUS-1_, an intrinsic chromosomal MBL gene from *M. odoratimimus* that encodes a MUS-1 subgroup 3a metalloenzyme [18]. Despite these findings, six MBL-positive isolates (three *C. indologenes* and three *S. maltophilia*) did not contain known intrinsic MBL genes *bla*_IND1–4_ or *bla*_L1_ [19], [20] as determined by PCR amplification. This might be due to the molecular heterogeneity of MBL genes in these species and requires further investigation.

Although the use of carbepenem antibiotics in food producing animals is prohibited in China and other countries, other β-lactam antibiotics such as penicillin and cephalosporins (cefradine, ceftiofur, and cefotaxime) have been extensively used in animal agriculture for the prevention and control of disease. Use of these non-carbepenem β-lactams in animal production may serve as a driving force for selecting MBL genes in bacteria. However, owing to the co-existence of MBL genes and other antimicrobial resistance genes, such as the *aphA6* in pAL-01 identified in this study, the possibility of cross-selection of MBL genes through use of other antimicrobials cannot be ignored. It should be noted that some carbapenemase-positive Gram-negative bacteria, including *Enterobacteriaceae*, with MIC values lower than 8 µg/mL might have been missed under the conditions used in this study.

Plasmid pAL-01 was successfully transferred from SGC-HZ9 to *E. coli* J53 using filter mating and to *E. coli* JM109 using electroporation, suggesting that this plasmid is mobile. The transformant and transconjugant were resistant to or showed decreased susceptibility to almost all tested β-lactams, but remained sensitive to other tested classes of antibiotics. These findings confirm the function of pAL-01 in conferring resistance to β-lactams, and suggest that the resistance to non-β-lactam antibiotics in SGC-HZ9 may be due to a chromosomal gene or plasmid other than pAL-01. Interestingly, the *bla*_NDM-1_-harboring plasmid was highly stable in *A. lwoffii* SGC-HZ9 and transformant JM109-SGC-HZ9, but was lost in transconjugant J53-SGC-HZ9 in the absence of antibiotic selection pressure. This finding is similar to that reported for a *bla*_NDM-1_-carrying plasmid in *A. baumannii* isolates of human origin [8]. Attempts were made to identify the incompatibility group of pAL-01 by replicon typing using the previously described PCR-based methods [21], [22], however, the replicon typing experiment yielded negative results and did not identified pAL-01 as a member of the known incompatibility groups for both *A. baumannii* and *Enterobacteriaceae*.

The location of the promoter sequence for *bla*_NDM-1_ was identified in a previous study by mapping the transcription start site [12]. The +1 transcription site of the gene was located 7 bp downstream of the putative −10 sequence. Furthermore, the −35 sequence overlaps with the 3′ end of the transposase and the right terminal repeat of IS*Aba125*. These results indicated that the expression of *bla*_NDM-1_ is driven by a promoter partly provided by IS*Aba125*. A similar structure was also found in studies of the aminoglycoside-modifying enzyme gene *aphA6*, for which the right inverted repeat of IS*Aba125* adjacent to *aphA6* provides the −35 sequence (TTGAAT), driving gene transcription [23], [24]. Because all of the known *bla*_NDM-1_ genes are associated with a partial or an intact IS*Aba125* (Fig. 2), and given that an intact IS*Aba125* has not been found in bacterial species other than *Acinetobacter*, it is possible that the *bla*_NDM-1_ gene may have originated in *Acinetobacter*, one of the most ubiquitous bacterial species in the environment. Like *bla*_NDM-1_, the *aphA6* gene was found to be located immediately upstream of IS*Aba125* (Fig. 2). However, transferring the *aphA6* gene (carried on pAL-01) to *E. coli* did not alter its susceptibility to kanamycin. It is therefore likely that *aphA6* in pAL-01 was not expressed (or expressed at an insignificant level) in the *E. coli* host. Alternatively, the high MIC value of kanamycin in SGC-HZ9 (64 µg/mL) might be conferred by other resistance determinants.

The results of this study confirm that *bla*_NDM-1_ and other MBL genes (*bla*_MUS-1_) are present in bacteria of food animal origin. These findings have an important implication for the dissemination of this resistance gene, as *Acinetobacter* spp. are highly prevalent in food-producing animals (chickens and pigs) and in the environment (soil, surface water, and sewage). It would be difficult to contain the spread of *bla*_NDM-1_ if it can be transferred to humans via the food chain. However, it is unclear whether the *bla*_NDM-1_-carrying *A. lwoffii* strain of food animal origin identified in this study represents a sporadic case or the early stage of a wide dissemination trend. Therefore, enhanced and continued efforts are needed to monitor the prevalence of *bla*_NDM-1_ in *Acinetobacter* spp. and other bacterial species of food animal origin.

## Materials and Methods

### Collection and identification of bacteria with low susceptibility to imipenem

A total of 396 samples were collected from eight chicken farms (n = 146, cloacal swabs), six duck farms (n = 50, cloacal swabs), one pig farm (n = 70, rectal swabs), and one pig slaughterhouse (rectal swabs n = 60 and nasal swabs n = 50 from the carcasses before evisceration, and lymph nodes swabs n = 20 from the carcasses after evisceration) in Shandong province, located in eastern China, from October to December 2010. Each sample was collected from a different animal and inoculated onto a brain heart infusion (BHI) agar plate containing 8 µg/mL imipenem and incubated at 30°C for 16–18 hours. All colonies on the culture plates were selected and identified using Gram staining and sequence analysis of the 16 s rDNA gene, using previously described primers [25]. Gram-negative bacteria were further confirmed by both the ATB 32GN and 32E identification systems (bioMérieux, Craponne, France). All collected strains were stored at −70°C in BHI supplemented with 15% glycerol until testing.

### Antibiotic Susceptibility Testing

Susceptibility of the bacterial isolates to 15 antibiotics was assessed using the broth micro-dilution method as set by the Clinical and Laboratory Standards Institute (CLSI) document M100-S18 (2008) [26].

### Phenotypic and molecular detection of MBL in Gram-negative bacteria

The imipenem-EDTA double-disc synergy test as well as E-test MBL strips (bioMérieux, Craponne, France) were used to screen for MBL production [27]. Strains were screened for known mobile MBL genes (*bla*_NDM-1_, *bla*_SIM-1_, *bla*_SPM-1_, *bla*_VIM_, *bla*_IMP_, *bla*_GIM-1_, *bla*_AIM-1_, and *bla*_DIM-1_) by PCR assay using previously described primers [2], [27], [28]. In addition, the chromosomal MBL genes (*bla*_IND1/2_ and *bla*_L1_) were also screened by PCR assay [29], [30]. Primers for mobile MBL gene *bla*_KHM_ and chromosomal MBL genes *bla*_MUS-1_, *bla*_TUS-1_, and *bla*_IND3/4_ designed in this study are listed in Table 2. All PCR amplicons were sequenced. The entire coding sequence of *bla*_NDM-1_ was synthesized by Beijing Genomics Institution (BGI, Beijing, China) according to the published sequence in plasmid pKpANDM-1 (accession number FN396876) and was used in the PCR assay as a positive control for *bla*_NDM-1_ [2].

**Table 2:** MBL gene primers designed in this study.

Primers	Sequence	GenBank accession No.
Mobile MBL gene		
KHM-F	ACGGATTAGTCGTGCTTGAT	AB364006
KHM-R	AATGCGTAGCCTGCTCTTC	
Chromosomal MBL gene		
MUS-1-F	ATCGTTCTGGTGGATTAGGTT	AF441286·1
MUS-1-R	CCTGTCATATCCCATTCATCA	
TUS-1-F	CGTTCGGGTGGACTTGATTAC	AF441287·1
TUS-1-R	GCGCTTTACGGCTTTGATTGT	
IND-3-F	TCAGTGCCCAGGTAAAAGAT	AF219133·2
IND-3-R	CCTCCACCTTTCCATTCATC	
IND-4-F	TGGCAGAAAACCCAGTATCAA	AF219135·2
IND-4-R	CGTTGCCTTTCCACTCGTC	

### Conjugation and transformation experiments

For conjugation, *A. lwoffii* SGC-HZ9 and *E. coli* J53 (azide resistant) were used as the donor and recipient, respectively, with selection on MacConkey medium containing sodium azide (100 µg/mL) and ceftazidime (16 µg/mL). Plasmid DNA was extracted from SGC-HZ9 with a QIAGEN Large-Construct Kit (Qiagen, Hilden, Germany). For transformation, the plasmid preparation was used to transform electro-competent *E. coli* JM109, and selection was performed on Luria Bertani (LB) agar plates containing ceftazidime (16 µg/mL). Transformants and transconjugants were further confirmed by specific PCR and Southern blotting.

### Southern blotting analysis

Transformants and transconjugants were further confirmed by specific PCR and Southern blotting. The sizes of the plasmids extracted from the original *A. lwoffii* strain and from the transformants were estimated by PFGE. Southern blotting was performed using a DIG High Prime DNA labeling and Detection Starter Kit (Roche, Basel, Switzerland) according to the manufacturer's instructions. The digoxigenin-labeled *bla*_NDM-1_-specific probe was prepared using primers (forward 5′-GGTTTGGCGATCTGGTTTTC-3′; and reverse 5′-CGGAATGGCTCATCACGATC-3′) that amplified a 621 bp region of the *bla*_NDM-1_ gene. Plasmids were extracted from *A. lwoffii* SGC-HZ9, transconjugant J53-SGC-HZ9, and transformant JM109-SGC-HZ9 using the QIAGEN Large-Construct Kit. Plasmid DNA was then separated by PFGE with the following conditions: 6.0 V/cm with an initial/final switch time of 5 s/50 s at an angle of 120° at 14°C for 10 hours. DNA was then transferred to a nylon membrane (Hybond N, Amersham, UK), which was hybridized with the prepared *bla*_NDM-1_-specific probe. Detection was performed using an NBT/BCIP color detection kit (Roche, Switzerland).

### Plasmid stability test

Plasmid stability was assessed by serial passage of SGC-HZ9, transconjugant J53-SGC-HZ9, and transformant JM109-SGC-HZ9 on antibiotic-free and ceftazidime-containing (16 µg/mL) LB media. Carriage of *bla*_NDM-1_ in these strains was assessed by inoculating the cultures of different passages on LB plates containing 16 µg/mL ceftazidime. Additionally, presence of *bla*_NDM-1_-carrying plasmids in cultures of different passages was confirmed by PCR assay. A plasmid was designated unstable if it was lost after three consecutive passages in antibiotic-free media.

### DNA cloning, sequencing, and analysis

Cloning was performed using vector pHSG298 with a kanamycin resistance marker (Takara, Dalian, China). Restriction endonucleases *Eco*RI, *Xba*I, and *Bam*HI (NEB, UK) were used to digest the plasmid DNA extracted from *A. lwoffii* SGC-HZ9 and transformant JM109-SGC-HZ9. Fragments were then separately ligated into pHSG298, which had been previously digested with the same enzymes. Transformation was carried out using electroporation into *E. coli* JM109. Transformants were selected on LB agar plates containing ceftazidime (16 µg/mL) and kanamycin (50 µg/mL). Recombinant plasmids containing *bla*_NDM-1_-positive inserts were confirmed by sequencing. The flanking regions of *bla*_NDM-1_ carrying restriction fragments in the plasmid of SGC-HZ9 were further sequenced using a modified random primer sequence walking strategy, as described previously [31]. The obtained sequences were annotated using the VectorNTI program (Invitrogen, Carlsbad; CA, USA), and sequence comparison was performed using BLASTP and BLASTN.
