# Correction: Identification of New Delhi Metallo-β-lactamase 1 in *Acinetobacter lwoffii* of Food Animal Origin

**DOI:** 10.1371/annotation/e33066ea-f500-4d34-8981-7cfd68497e73

**Published:** 2013-07-15

**Authors:** Yang Wang, Congming Wu, Qijing Zhang, Jing Qi, Hebing Liu, Yu Wang, Tao He, Licai Ma, Jing Lai, Zhangqi Shen, Yuqing Liu, Jianzhong Shen

The number of the grant from the National Natural Science Foundation of China is incorrect. The correct grant number is: U1031004.

